# Coauthorship and Institutional Collaborations on Cost-Effectiveness Analyses: A Systematic Network Analysis

**DOI:** 10.1371/journal.pone.0038012

**Published:** 2012-05-29

**Authors:** Ferrán Catalá-López, Adolfo Alonso-Arroyo, Rafael Aleixandre-Benavent, Manuel Ridao, Máxima Bolaños, Anna García-Altés, Gabriel Sanfélix-Gimeno, Salvador Peiró

**Affiliations:** 1 Centro Superior de Investigación en Salud Pública (CSISP), Valencia, Spain; 2 Fundación Instituto de Investigación en Servicios de Salud, Valencia, Spain; 3 Division of Pharmacoepidemiology and Pharmacovigilance, Spanish Medicines and Healthcare Products Agency (AEMPS), Madrid, Spain; 4 Unidad de Información e Investigación Social y Sanitaria-UISYS, Universidad de Valencia-CSIC, Valencia, Spain; 5 Departamento de Historia de la Ciencia y Documentación, Facultad de Medicina y Odontología, Universidad de Valencia, Valencia, Spain; 6 Instituto Aragonés de Ciencias de la Salud (I+CS), Zaragoza, Spain; Hungarian Academy of Sciences, Hungary

## Abstract

**Background:**

Cost-Effectiveness Analysis (CEA) has been promoted as an important research methodology for determining the efficiency of healthcare technology and guiding medical decision-making. Our aim was to characterize the collaborative patterns of CEA conducted over the past two decades in Spain.

**Methods and Findings:**

A systematic analysis was carried out with the information obtained through an updated comprehensive literature review and from reports of health technology assessment agencies. We identified CEAs with outcomes expressed as a time-based summary measure of population health (e.g. quality-adjusted life-years or disability-adjusted life-years), conducted in Spain and published between 1989 and 2011. Networks of coauthorship and institutional collaboration were produced using PAJEK software. One-hundred and thirty-one papers were analyzed, in which 526 authors and 230 institutions participated. The overall signatures per paper index was 5.4. Six major groups (one with 14 members, three with 7 members and two with 6 members) were identified. The most prolific authors were generally affiliated with the private-for-profit sector (e.g. consulting firms and the pharmaceutical industry). The private-for-profit sector mantains profuse collaborative networks including public hospitals and academia. Collaboration within the public sector (e.g. healthcare administration and primary care) was weak and fragmented.

**Conclusions:**

This empirical analysis reflects critical practices among collaborative networks that contributed substantially to the production of CEA, raises challenges for redesigning future policies and provides a framework for similar analyses in other regions.

## Introduction

In recent years, rising demand and constrained resources–enhanced by the recent economic and financial crisis–is making cost-effectiveness one of the most important goals in healthcare. Cost-effectiveness analysis (CEA), sometimes part of the health technology assessment process, involves the comparisons of alternative options in terms of their costs and their outcomes. CEA has been promoted as an important research methodology for determining the efficiency of healthcare technology and guiding societal decision-making on the financing of public healthcare services. At the same time, and as was the case with clinical research [1]–[4], conflicts of interest due to the influence of the pharmaceutical and medical technology industries is emerging as a critical issue in economic research [5], [6].

Although there is some controversy about the role of CEA in decision-making at the level of healthcare administrations and individual hospitals, various national healthcare systems are now using economic evidence to make system-level decisions about which interventions to fund from collective resources [7], with Australia, New Zealand, the United Kingdom and Canada pioneering this scheme [8], [9]. In other countries, the regulatory and institutional framework has so far not helped the introduction of CEAs and their application to the decisions of public health funding. Such is the case in Spain, where the 2006 Pharmaceuticals and Healthcare Products Law [10] avoided any explicit reference to cost-effectiveness. Recently, the 2011 package of measures to reduce fiscal deficit incorporated the possibility of using economic criteria in the price-fixing decisions, but uncertainties about the specific role CEA will play in the market access of new medicines and healthcare products still remain [11], [12], including the scientific capacity of the research structures to produce the cost-effectiveness studies that the Spanish healthcare system needs.

A comprehensive systematic analysis of current scientific activity and practice is critical to understanding the maturity and growth of any research area [13]. Social network analysis is grounded in the assessment of empirical data and can provide an appropriate approach to identify researchers, groups and organizations. It also offers highly interesting information to understand the structure and nature of relationships and interaction within a scientific community [14]–[17]. This study aims to describe and characterize collaborative patterns on CEA conducted over the past two decades in Spain, applying techniques from social network analysis and bibliometrics.

**Table 1:** Characteristics of cost-effectiveness analyses, 1989–2011 (n=131).

Characteristics	Number	% of total
**Year of publication**		
1989–1995	3	2.3
1996–2000	8	6.1
2001–2005	19	14.5
2006–2011	101	77.1
**Journals containing 3 or more papers**		
PharmacoEconomics - Spanish Research Articles	10	7.6
Health Technology Assessment reports	9	6.8
*Revista Española de Economía de la Salud*	8	6.1
*Gaceta Sanitaria*	7	5.3
European Journal of Health Economics	4	3.0
Current Medical Research and Opinion	4	3.0
Clinical and Translational Oncology	4	3.0
*Revista de Neurología*	4	3.0
Value in Health	4	3.0
Osteoporosis International	3	2.3
PharmacoEconomics	3	2.3
*Revista Española de Salud Pública*	3	2.3
Vaccine	3	2.3
*Vacunas*	3	2.3
**Journal type**		
Medical and clinical	77	58.8
Economics, health policy and healthServices research	54	41.2
**Origin of publication**		
Local/sub-national level	67	51.1
International	64	48.9
**JCR 2010 impact factor, quartiles (Q)**		
Q1	29	22.1
Q2	23	17.6
Q3	18	13.7
Q4	13	9.9
None	48	36.6

JCR: Journal Citation Report, year 2010.

**Figure 1:**
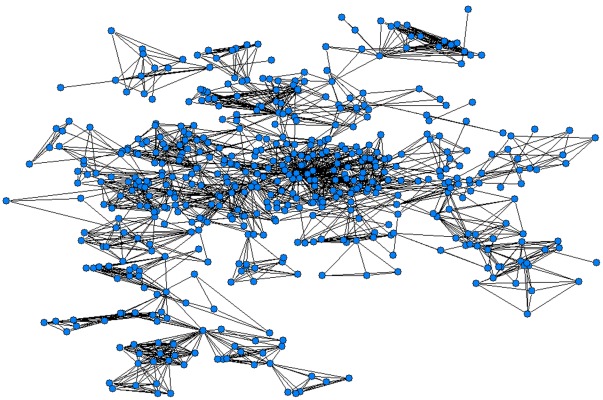
Full network of coauthorship. Note: Each circle represents one author and each line connecting two of them reflects the presence of at least one paper they have co-authored.

**Figure 2:**
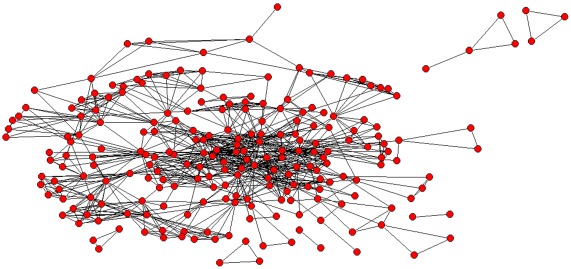
Full network of institutional collaboration. Note: Each circle represents one institution and each line connecting two of them reflects the presence of at least one paper they have co-authored.

## Methods

### Comprehensive Systematic Review of CEAs

We updated a previous comprehensive literature review which had examined CEAs conducted in Spain within the period 1983–2008 [18], [19], adding the studies published until September 2011. Briefly, this systematic review was conducted in PubMed/MEDLINE, SCOPUS, ISI Web of Knowledge, Databases of the Centre for Reviews and Dissemination (CRD), as well as *Índice Médico Español* (IME) and *Índice Bibliográfico Español en Ciencias de la Salud* (IBECS). Medical subject heading (MeSH) descriptors were used distributed into two blocks: economic evaluation and Spain. In addition, hand searches were carried out for reports from health technology assessment agencies and papers in specialized local journals partially included in the abovementioned databases. Reviews, editorials and abstracts of congresses were excluded.

Our selection of articles was based on the criteria of full economic evaluation (evaluations where both costs and health outcomes have been measured) of healthcare interventions. In particular, this study focuses on CEAs that use outcomes as a time-based summary measure of population health that captures both prolongation and health-related quality of life (e.g. quality-adjusted life-years [QALYs] or disability-adjusted life years [DALYs]), also known as “cost-utility analyses”. We selected this type of CEA because many decision-makers and researchers have recommended the QALY/DALY framework as the standard reference for cost-effectiveness [20], [21].

**Table 2:** Ranking of most productive authors and their collaborative patterns.

Ranking	Author	Affiliation (type)	Papers	Signatures^a^	Authors/paper index	Collaborators^a^
**1**	**Brosa-Riestra M**	**Oblikue Consulting (consulting firm)**	**15**	**98**	**6.5**	**73**
**2**	**Rubio-Terrés C**	**HERO Consulting (consulting firm)**	**11**	**56**	**5.1**	**40**
**3**	**Rodríguez-Barrios JM**	**Medtronic Ibérica S.A. (pharmaceutical** **industry)**	**8**	**59**	**7.4**	**46**
**4**	**Casado MA**	**PORIB (consulting firm)**	**7**	**36**	**5.1**	**20**
5	Mar J	Hospital Alto Deba (hospital)	7	25	3.6	17
6	López-Bastida J	Servicio Canario de Salud (healthcare administration)	6	28	4.7	21
**7**	**Crespo C**	**Oblikue Consulting, Universidad Autónoma** **de Barcelona (consulting firm, academia)**	**5**	**48**	**9.6**	**38**
**8**	**Oyagüez I**	**PORIB (consulting firm)**	**5**	**30**	**6.0**	**21**
**9**	**Díaz-Cerezo S**	**Pfizer S.A. (pharmaceutical industry)**	**5**	**30**	**6.0**	**19**
**10**	**Rejas-Gutiérrez J**	**Pfizer S.A. (pharmaceutical industry)**	**5**	**27**	**5.4**	**17**
11	Buti M	Hospital Vall d’Hebrón (hospital)	5	25	5.0	14
**12**	**Dilla T**	**Lilly S.A. (pharmaceutical industry)**	**4**	**22**	**5.0**	**15**
13	Haro JM	Fundació Sant Joan de Deu (hospital)	4	24	6.0	14
14	Jönsson B	Stockholm School of Economics (academia)	4	37	9.2	33
**15**	**Badía X**	**IMS Health S.A. (consulting firm)**	**3**	**18**	**6.0**	**15**

a In papers where the author has participated. Authors in bold indicate authors affiliated with private-for-profit sector.

**Figure 3:**
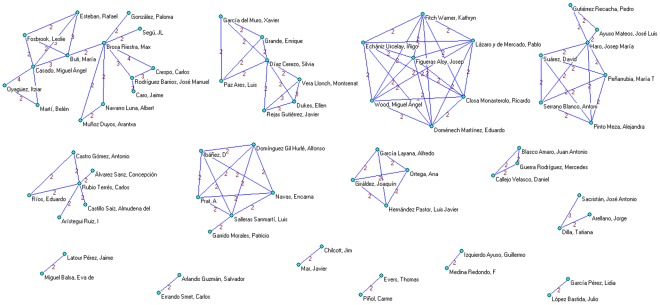
Clusters of authors applying a threshold of two or more papers signed in coauthorship.

For each paper selected, we recorded the year of publication, the journal title, country of publishing editor (local or international), the name and surnames of the authors as well as their institutional affiliation. Journals were classified as medical and clinical journals (e.g. Current Medical Research and Opinion, *Revista de Neurología*, Clinical and Translational Oncology) or economics, health policy, and health services research journals (e.g. PharmacoEconomics, *Gaceta Sanitaria*, European Journal of Health Economics, reports of Health Technology Assessment).

The journals’ impact factor was obtained from the 2010 Journal Citation Reports (JCR) and was categorized into quartiles. We used the expanded Science Citation Index (ISI Web of Knowledge) to determine the extent to which each study has been cited in other publications. To homogenize authorship, we checked the signatures with which an individual appeared in two or more different forms, using coincidence in authors’ places of work as the basic criterion for normalization. In the case of institutions, we have unified the different variants to match the name recorded in the Spanish National Catalogue of Hospitals, the National Registry of Universities and public directories of institutions. Similarly, given that institutional names in many records included two or more institutions (e.g. university hospitals and academia; consulting firms and academia), we have proceeded to distinguish between these signatures by recording as many signatures as individual macroinstitutions could be identified for each bibliographic record. With this information we constructed a Microsoft Access database.

### Indicators and Networks of Coauthorship and of Institutional Collaboration

We use the term *coauthorship* to refer to joint authorship of a scientific paper by 2 individuals, and *institutional collaboration* to refer to joint authorship by different institutions. *Intensity of collaboration* or *threshold* refers to the figure used to form clusters of authors and institutions, referring to the frequency of coauthorship between pairs of authors or of collaboration between institutions, and reflects a criterion to label identifiable clusters as *research groups*.

Collaboration between authors is portrayed by calculating the number of papers, signatures, collaborations, the index of signatures per paper or collaboration index, which is the mean number of signatures per paper; and the index of authors per paper (mean number of authors per paper considering only the different authors).

To construct coauthorship networks, we identified all combinations of pairs of authors for each paper. The number of coauthorships for each paper is related to the number of authors as it is equal to m!/(m-n)!n!, where *m* is the number of individual authors and *n* the number of elements in the groups constructed. Once coauthorship was quantified, we further established a threshold of 2 or more collaborations between pairs of authors to reduce the number of nodes and links that would prevent a clear view of the network and thus centre analysis on the more intense coauthorship relationships. The same approach was applied to institutional authorship to construct the network of interinstitutional collaboration, although in this case we applied a threshold of at least 3 collaborations. We used PAJEK software [22] to analyse indicators and construct social networks.

**Table 3:** Ranking of most productive institutions and their collaborative patterns.

Ranking	Institution (type)	Papers	Signatures	Collaborations	Collaborators
**1**	**Oblikue Consulting (consulting firm)**	**14**	**81**	**14**	**50**
**2**	**Pfizer S.A. (pharmaceutical industry)**	**14**	**62**	**14**	**32**
3	Universidad Autónoma de Barcelona (academia)	12	68	11	43
**4**	**HERO Consulting (consulting firm)**	**10**	**34**	**10**	**18**
**5**	**Medtronic Ibérica S.A. (pharmaceutical industry)**	**8**	**47**	**8**	**30**
6	Hospital Vall d’Hebron (hospital)	8	39	7	29
7	Hospital Clínic de Barcelona (hospital)	8	29	7	15
8	Hospital Universitario 12 de Octubre (hospital)	7	28	6	20
9	Complejo Universitario San Carlos (hospital)	6	35	6	27
10	Complejo Universitario La Paz (hospital)	6	34	6	23
11	Hospital Alto Deba (hospital)	6	21	6	13
**12**	**Pfizer Inc. USA (pharmaceutical industry)**	**5**	**25**	**5**	**14**
13	Fundació Sant Joan de Déu (hospital)	4	18	4	9
**14**	**Merck and Co., Inc. (pharmaceutical industry)**	**3**	**16**	**3**	**13**
15	Hospital Universitari de Bellvitge (hospital)	2	22	15	2

Institutions in bold indicate private-for-profit sector.

**Figure 4:**
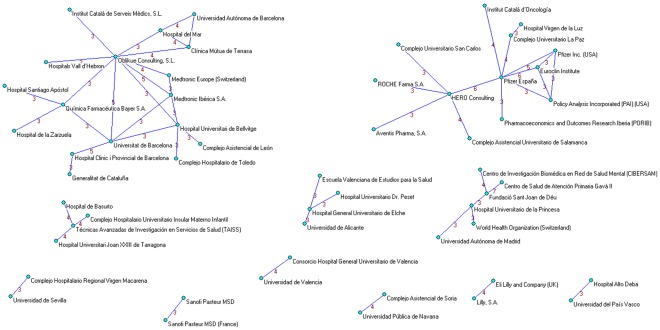
Clusters of institutions applying a threshold of three or more papers signed in institutional collaboration.

**Figure 5:**
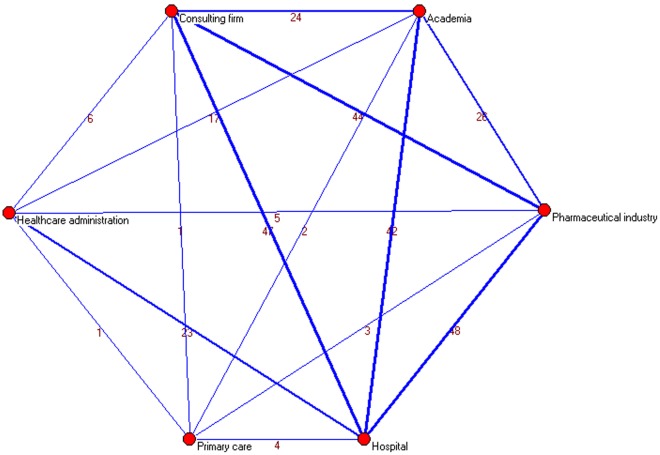
Institutional collaboration network.

## Results

Data on 131 articles published between 1989 and 2011 was judged to contain eligible CEAs and became part of the analysis. Ninety-nine percent of the papers were signed in collaboration by two or more authors; 710 signatures were identified for an overall collaboration index of 5.4 signatures per paper.

The number of publications increased exponentially over the study period (Table 1). More than three-quarters of the CEAs were carried out during 2006–2011. PharmacoEconomics – Spanish Research Articles was the journal that published the greatest number of manuscripts (n=10; 7.6%), followed by reports for the Health Technology Assessment agencies (n=9; 6.8%), *Revista Española de Economía de la Salud* (n=8; 6.1%) and *Gaceta Sanitaria* (n=7; 5.3%). Nearly 40% (n=52) of the CEAs were published in journals within the first and second quartile of their speciality. By journal type, 59% (n=77) of the studies were disseminated in medical and clinical literature. Figures 1 and 2 show the structure of the full networks, in which 526 authors and 230 institutions participated. The network’s size (given by the number of nodes) is discernable and not visually dense (given by the number of connections).

**Table 4:** List of most cited cost-effectiveness analyses (with at least one Spanish author).

Ranking	Authors	Title	Journal	Year	Citations
1	**Mar J**, Rodríguez-Artalejo F.	Which is more important for the efficiency of hypertensiontreatment: hypertension stage, typeof drug or therapeutic compliance?	*Journal of* *Hypertension*	2001	53
2	**Buti M**, **Casado MA**, Fosbrook L, Wong JB, Esteban R.	Cost-effectiveness of combination therapy for naive patientswith chronic hepatitis C	*Journal of* *Hepatology*	2000	47
3	Añón JM, García de Lorenzo A, Zarazaga A, Gómez Tello V,Garrido G.	Mechanical ventilation of patients on long-term oxygentherapy with acute exacerbations of chronic obstructivepulmonary disease: prognosis and cost-utility analysis	*Intensive Care Medicine*	1999	39
4	**Mar J**, Rueda JR, Durán Cantolla J, Schechter C, Chilcott J.	The cost-effectiveness of nCPAP treatment in patientswith moderate-to-severe obstructive sleep apnoea	*European Respiratory Journal*	2003	36
5	**Mar J**, Begiristáin JM, Arrazola A.	Cost-effectiveness analysis of thrombolytic treatmentfor stroke	*Cerebrovascular Diseases*	2005	31
6	Pereira A.	Cost-effectiveness of transfusing virus-inactivated plasmaInstead of standard plasma	*Transfusion*	1999	29
7	Latour-Pérez J, Navarro-Ruiz A, Ridao-López M, Cervera-Montes M.	Using clopidogrel in non-ST-segment elevation acutecoronary syndrome patients: a cost-utility analysisin Spain	*Value in Health*	2004	24
8	Plans-Rubió P, Garrido-MoralesP, Salleras Sanmartí L.	The cost-effectiveness of pneumococcal vaccinationin Catalonia	*Revista Española de* *Salud Pública*	1995	24
9	Rodríguez MJ, **Díaz-Cerezo S**,Vera Llonch M, Dukes E,**Rejas-Gutiérrez J**.	Cost-effectiveness analysis of pregabalin versus gabapentinin the management of neuropathic pain due to diabeticpolyneuropathy or post-herpetic neuralgia	*Current Medical* *Research and Opinion*	2007	19
10	Rutten Van Mölken MP,Oostenbrink JB, Miravitlles M,Monz BU.	Modelling the 5-year cost effectiveness of tiotropium,salmeterol and ipratropium for the treatment of chronicobstructive pulmonary disease in Spain	*European Journal of* *Health Economics*	2007	19

Authors in bold indicate prolific authors according to number of publications (Table 1).

We identified 14 authors who published more than 3 papers. The most prolific authors (Table 2) were Brosa-Riestra and Rubio-Terrés with 15 and 11 papers, respectively. Two thirds of the 14 most prolific authors were affiliated with consulting firms and/or the pharmaceutical and medical technology industries. Applying a threshold of two or more papers signed in coauthorship (Figure 3), we identified 15 clusters of authors. Of them, 6 major groups (one with 14 members, three with 7 members and two with 6 members) were identified; 4 of these 6 most collaborative authors’ clusters included at least one industry-affiliated authorship.

Institutional productivity was headed by Oblikue Consulting and Pfizer S.A. with 14 papers each (Table 3). Next came the *Universidad Autónoma de Barcelona* and HERO Consulting with 12 and 10, respectively, and *Medtronic Ibérica S.A.*, *Hospital Vall d’Hebró* and *Hospital Clínic de Barcelona* with 8 each. Applying a collaboration threshold of at least 3 papers signed with interinstitutional collaboration, we identified 11 clusters comprising by a total of 56 institutions (Figure 4 and 5). The private-for-profit sector mantains broad collaborative networks including public hospitals and academia. Collaboration within the public sector (e.g., healthcare administration and primary care) was weak and fragmented.

Among the 34 authors who published the top-10 most cited CEAs (Table 4), only 5 (13%) are among the top-15 most prolific authors. The article by Mar and Rodríguez-Artalejo [23] was the most frequently cited. Only one author appeared more than once in the top-10 most cited CEAs list, with 3 papers [23]–[25] attracting 120 citations. Since older articles are more likely to generate more citations, only three out of the top-10 were published in the second half of the 2000s.

## Discussion

Our paper synthesizes empirical data on the development of CEAs in Spain using social network analysis. This methodology allowed us to identify the most productive authors and institutions, as well as the structure and patterns of collaboration that have published papers over a 22-year period. Perhaps the most significant finding is that networks of scientific collaboration reveal a discernable and limited scientific community, with most individuals having only a few coauthors, whereas a few firms have many collaborations. Remarkably, the scientific community captured by the network analysis is centred on a nucleus of authors from private-for-profit companies (consulting firms and the pharmaceutical industry) and affiliated collaborators from hospitals and academia.

Very few studies have reviewed CEAs using social network analysis, and although not directly comparable with our analysis, there are aspects worthy of comment. The recent study by Greenberg et al.[26], a CEA review of the English-language articles indexed in PubMed/MEDLINE since 2006, observed that the most prolific authors were affiliated with renowned academic and hospital centres but did not find any substantial private-for-profit (e.g. pharmaceutical industry) relationships across groups. These differences with our results are striking and probably part of the different public and academic commitment to health services research and CEA studies in Spain. However, the inclusion of all types of studies –regardless of language or publication status and indexing in bibliographic databases– may be a contributing factor to these differences: more than one-third of our selected articles were published in non-indexed journals with clear links to commercial third parties, while Greenberg et al [26] used only PubMed/MEDLINE indexed papers.

There are several possible explanations for our findings. Industry funds an increasing proportion of medical research [27]. Cost-effectiveness is aimed at reducing resource allocation and may have significant implications on public decisions, therefore industry-funded CEAs may be part of promotional strategies aimed at demonstrating that new products are cost-effective despite their higher price and at enhancing market access activities. Other important health policy questions with no commercial connections would not be supported by the private sector, and therefore the Spanish CEA literature is probably reflecting the research priorities of private industry rather than informing policy-makers on efficient ways to afford the health needs of the population [18]. Additionally, a growing body of literature has been drawing attention to the fact that industry-sponsored studies are more likely than non-industry sponsored studies to report results and conclusions favouring the sponsor’s product or to report more favourable cost-effectiveness ratios [5], [6], [28]–[34]. In fact, negative industry-sponsored CEA studies are extremely infrequent. Overall, we are facing a literature with a high risk of bias. The high prevalence of studies conducted or funded by commercial third parties could lead to a reduction in the credibility and actual use of this type of research [35]. In fact, few decision-makers anywhere in the industrialized countries seem to rely on these types of analyses in their decisions [36]. Exceptions include countries with well-established public agencies, such as NICE in the United Kingdom [37].

In Spain, the central government is the main decision-maker in pricing and reimbursement related to new medicines, but traditionally there have been no national requirements related to the cost-effectiveness of new medicines. A direct consequence of this situation is that the public sector may have indeed lost control of the CEA research agenda. Despite recent policies of promoting interinstitutional collaboration and public network research [38], we have only detected small clusters in which the institutions involved are public ones, denoting a clear underrepresentation of the healthcare administration. Similarly, few authors from the public sector seem to produce these types of analyses, and those who do so are scientifically isolated (although they published the most influential studies). This context is changing with the introduction of new policy measures and the creation of new policy-oriented committees, but cutbacks in funding health research in Spain will not facilitate the development of an independent publicly funded CEA research network.

Our study has several limitations. First, although the scientific production analyzed has been drawn from an exhaustive, comprehensive review of original research of cost-effectiveness studies, it is possible that the search missed some articles with relevant elements or that some studies conducted by manufacturers may not have been published. The analyses inevitably represent only a first overview of research in this area. Furthermore, we restricted our analysis to CEAs that measured health outcomes with QALYs or DALYs, and therefore there may be researchers (and institutions) who do not appear because their publications are not reflected in the networks. It would be interesting to explore whether the use of alternative outcome measurements results in similar patterns of collaborations. Our analysis was also limited in scope, focusing as it does only on original cost-effectiveness research. Undoubtedly, there are other important reports (including reviews of economic evaluations) that also merit consideration in decision-making. Second, the networks identified primarily reflect recent publications (more than three-quarters of the papers were published in the last 5 years). Given the dynamic nature of research, other opportunities for further research include examining the evolution of the identified networks over time (e.g. by means of longitudinal social network analysis). Third, as in many other bibliometric analyses, the importance of normalizing the names of authors and institutions is fundamental to avoiding errors caused in recognizing variations in the name of a single author, or considering scientific production of 2 or more different authors with the same names as belonging to one individual. Nevertheless, we conducted a careful manual validation of the bibliographic references to avoid these errors. Fourth, in a context of scientific literature dominated by market orientation, we can expect some presence of ghostwriters and honorary authorships [39]–[41], there being some indirect evidence of these practices [42]. Ghostwriters and honorary authorships could distort the real CEA networks, reducing the importance of private firms and falsely increasing the academic presence. Finally, the analysis of coauthorship and institutional collaborations for constructing networks has only recently become an object of study in health services research, and uniform criteria to identify communities or research groups within previously constructed networks are generally lacking. This may explain some of the differences in the observed patterns of authorship and institutional collaboration with other studies [26], especially because reports identified outside widely-used databases and readily accessible journals may be of lower quality than studies that are easier to access [43].

In summary, our study reflects current critical practices among collaborative networks that contribute substantially to the production of CEAs in Spain, raises challenges for redesigning future policies and provides a framework for similar analyses in other regions. We believe there is an urgent need to implement cost-effectiveness criteria together with clinical effectiveness and safety to assess what it may be realistic to pay for health gains. In Spain, the credibility of current fiscal adjustments and the solvency of the National Health System will depend primarily on the ability to evolve rapidly towards the selective funding of healthcare technologies, including new price-fixing schemes to ensure value-for-money for new medicines, and therefore evidence-based policies. Rational structural changes are required in the current procedures. Moreover, as the government and local healthcare administrations increasingly have a role in decisions related to cost-effectiveness, a well-connected and coordinated network becomes more important. Incentivizing the sustained involvement of researchers in the public health sector potentially addresses the lack of technical capacities and needs, as well as facilitating cooperation between researchers and policy-makers [44]. Complementary options might include the establishment of a board or independent agency that includes policy-makers as well as researchers to develop and/or approve a common cost-effectiveness research agenda that is relevant to health policy.
